# Astrocytes Modulate Local Field Potential Rhythm

**DOI:** 10.3389/fnint.2015.00069

**Published:** 2016-01-11

**Authors:** Shivendra G. Tewari, Vladimir Parpura

**Affiliations:** ^1^Molecular and Integrative Physiology, University of MichiganAnn Arbor, MI, USA; ^2^Department of Neurobiology, University of Alabama at BirminghamBirmingham, AL, USA

**Keywords:** astrocytes, contextual memory, computational neuroscience, hippocampus, tripartite synapse

Ever since memory deficits were characterized in patient H.M. (Scoville and Milner, 1957), it has become clear that the hippocampal region is an important player in memory acquisition and retrieval. Not to mention that memory is of paramount importance for maintaining relationships, achieving personal and professional goals, and carrying out even simple day-to-day activities. Scientists have been interested in understanding the basic mechanism by which our brain perceives and processes information, stores, and then later on (when required) retrieves it. Sometimes it is not possible to retrieve relevant information unless we recall events (context) associated with the memory trace. This begs a few questions: Why is the memory retrieval depressed or enhanced under some conditions? Is this a function of long-term synaptic facilitation or depression? If so, what are the processes that underlie synaptic facilitation or depression?

With the technological advancements made in the past 3–4 decades, it has been possible to start addressing these questions by recording spiking activity from a single neuron or a population of neurons (i.e., Local Field Potentials, LFPs) during memory acquisition and retrieval (Fried et al., 1997). It is widely believed that LFPs are the spatially weighted averages of synaptic currents (Kraskov et al., 2007). These synaptic currents are in some cases facilitated even though the amplitude and frequency of stimulating protocol is unchanged—a phenomenon widely known as long-term potentiation (Bliss and Collingridge, 1993). Synchronized synaptic activity within specialized brain regions, recorded as LFPs, have been reported during events of memory acquisition and retrieval (Eldridge et al., 2000; Kraskov et al., 2007) but the fundamental processes involved in this coordinated activity of neurons are still mainly unknown (Uhlhaas and Singer, 2006).

Astrocytes have emerged as an important player in synaptic plasticity in the past two decades. These glial cells were considered merely a “glue” for the most part of the last century till it was demonstrated that astrocytes respond to glutamate, a neurotransmitter released by excitatory neurons, by increasing their cytosolic calcium concentration (Cornell-Bell et al., 1990) and providing a feedback to neuron-neuron signaling by, for example: (1) secreting glutamate and activating metabotropic glutamate receptors (mGluRs) on pre-synaptic neurons (Perea and Araque, 2007) and/or (2) secreting D-serine and modulating N-methyl-D-Aspartate receptors (NMDARs) on post-synaptic neurons (Henneberger et al., 2010). Moreover, individual astrocytes are known to have fine processes that can enwrap asymmetrical (mainly excitatory) synapses (Fellin et al., 2006). Thus, strategically, astrocytes are ideally positioned to receive signal and relay it onto a small population of neurons during specific cognitive tasks.

The majority of experiments conducted so far have suggested that astrocytes contribute to short-term (Perea and Araque, 2007; Sibille et al., 2014, 2015) and long-term potentiation of individual synapses (Perea and Araque, 2007; Henneberger et al., 2010; Shigetomi et al., 2013). Several mathematical models have been developed (Nadkarni and Jung, 2007; Volman et al., 2007; De Pittà et al., 2009; Wade et al., 2011; Tewari and Majumdar, 2012b) to explore the tripartite synapse, a conventional neuron-neuron synapse with an add-on of a peri-synaptic astrocyte. However, relatively few experiments on hippocampal slices (Fellin et al., 2004; Sasaki et al., 2014) are available which report on the effect of astrocyte signaling on neuronal synchrony. These experiments hint at a role of astrocytes in tuning LFPs during events of memory acquisition and/or retrieval (see Table 1 for a list of LFP rhythms and associated cognitive function).

**Table 1:** **List of different LFP types and their proposed behavioral function**.

**Rhythm type**	**Frequency**	**Proposed role**
Delta oscillations	1–4 Hz	Attention, salience detection, and subliminal perception (Knyazev, 2012).
Theta oscillations	4–12 Hz	Active behavior, spatial navigation, memory, and sleep (Tort et al., 2008).
Gamma oscillations	30–100 Hz	Sensory binding, selective attention, neuronal assembly, information transmission and storage (Tort et al., 2008).
Sharp wave ripples	120–200 Hz	Consummatory behavior, immobility, slow wave sleep (Buzsáki et al., 2003).

As stated earlier, there are several mathematical models which quantify the effect of astrocytes at a single synapse. However, there are not many mathematical models which attempt at integrating the effect of astrocytes on a network of synapses and possible LFP rhythm generation and its modulation. One such mathematical model was developed by Tewari and Parpura (2013) that simulated the effect of astrocyte signaling on neuronal synchrony and LFP generation using published biophysics-based models of neurons and astrocytes. Briefly, four CA1 pyramidal neurons (with recurrent synapses) were stimulated by a single CA3 pyramidal neuron with one astrocyte present in the *stratum oriens* wrapping the CA3-CA1 synapses. The mathematical model of pyramidal neurons was the branching dendrite model (Traub et al., 1991, 1994) and astrocyte wrappings were modeled using the tripartite synapse model (Tewari and Majumdar, 2012a,b). LFP computation of their small CA1 network suggested astrocyte-dependent delta oscillations (Tewari and Parpura, 2013). Otherwise, the modeled neuronal network exhibited a theta rhythm. It should be noted that CA3 pyramidal neuron firing is essential for a recall of memory related patterns (Makara and Magee, 2013). Therefore, recurrent CA3 pyramidal neuron activity may affect the rhythm of CA1 LFP during recall of memory related patterns. Of note, the sole existing model (Tewari and Parpura, 2013) did not account for these recurrent connections within CA3 pyramidal neurons.

It is interesting that a recent experimental study has also demonstrated that the ablation of astrocytic vesicular release leads to the impairment of cognitive abilities in awake mice (Lee et al., 2014). More specifically, Lee et al. (2014) observed astrocyte-dependent tuning of cortical neurons to gamma rhythm, a result which appears to be in contrast with the model predictions of Tewari and Parpura (2013). However, the differences can be reconciled by: (1) the presence of neocortical interneurons known to oscillate at gamma frequency (Bartos et al., 2007) and/or (2) the size of network used to compute field potentials. It is quite possible that different brain regions may have different firing patterns in response to a given task. An alternate explanation for this difference in LFP rhythm observed in the model (Tewari and Parpura, 2013) and data (Lee et al., 2014) can be merely due to the choice of parameters used in the modeling approach; e.g., number of concurrent CA1 synapses, density of extra-synaptic NMDAR current etc.

To summarize, it has now become quite clear, from experiments and modeling simulations, that astrocytes do play an important role in modulating mammalian cognitive and behavioral abilities. But whether their contribution is specific to certain cognitive tasks than others remains to be seen. Availability of such information could be beneficial in treating conditions that pertain to memory deficit disorders.

## Funding

We acknowledge the support by the National Institutes of Health (The Eunice Kennedy Shriver National Institute of Child Health and Human Development award HD078678; and National Institute of General Medical Sciences award P50-GM094503).

### Conflict of interest statement

The authors declare that the research was conducted in the absence of any commercial or financial relationships that could be construed as a potential conflict of interest.
